# Mass Spectrometry Offers Insight into the Role of Ser/Thr/Tyr Phosphorylation in the Mycobacteria

**DOI:** 10.3389/fmicb.2016.00141

**Published:** 2016-02-12

**Authors:** Bridget Calder, Claudia Albeldas, Jonathan M. Blackburn, Nelson C. Soares

**Affiliations:** Applied and Chemical Proteomics Group, Medical Biochemistry Division, Faculty of Health Sciences, Institute of Infectious Diseases and Molecular Medicine, University of Cape TownCape Town, South Africa

**Keywords:** post-translational modification, phosphorylation, mycobacteria, tuberculosis, proteomics, phosphoproteomics, mass spectrometry, virulence

## Abstract

Phosphorylation is a post translational modification which can rapidly regulate biochemical pathways by altering protein function, and has been associated with pathogenicity in bacteria. Once engulfed by host macrophages, pathogenic bacteria are exposed to harsh conditions and must respond rapidly in order to survive. The causative agent of TB, *Mycobacterium tuberculosis*, is unusual amongst the bacteria because it can survive within the host macrophage for decades in a latent state, demonstrating a remarkable capacity to successfully evade the host immune response. This ability may be mediated in part by regulatory mechanisms such as ser/thr/tyr phosphorylation. Mass spectrometry-based proteomics has afforded us the capacity to identify hundreds of phosphorylation sites in the bacterial proteome, allowing for comparative phosphoproteomic studies in the mycobacteria. There remains an urgent need to validate the reported phosphosites, and to elucidate their biological function in the context of pathogenicity. However, given the sheer number of putative phosphorylation events in the mycobacterial proteome, and the technical difficulty of assigning biological function to a phosphorylation event, it will not be trivial to do so. There are currently six published phosphoproteomic investigations of a member of mycobacteria. Here, we combine the datasets from these studies in order to identify commonly detected phosphopeptides and phosphosites in order to present high confidence candidates for further validation. By applying modern mass spectrometry-based techniques to improve our understanding of phosphorylation and other PTMs in pathogenic bacteria, we may identify candidates for therapeutic intervention.

## Introduction

Proteins are the bioactive molecule in the cell, and contribute to survival, growth, and reproduction by interacting with each other, and with metabolites, lipids, nucleic acids and carbohydrates, and catalyzing biological reactions (Nørregaard Jensen, 2004). Protein biosynthesis and degradation are tightly regulated by complex biochemical systems in response to the changing needs of the cell. However, there is an additional mechanism which allows cells to respond rapidly and efficiently to the external and internal conditions. Post translational modification (PTM) by selective covalent processing of proteins—by proteolytic cleavage or the addition of a modifying group—can drastically alter the properties of a protein (Mann and Jensen, 2003; Mijakovic, 2010; Stülke, 2010). Post translational modifications add a layer of complexity to both bacterial and eukaryotic mechanisms of adaptation to the surrounding environment.

Modern mass spectrometry (MS) has enabled us to perform high throughput analysis of PTMs. Since modified proteins usually occur at low abundance, an enrichment process is typically carried out for a specific PTM prior to MS analysis (Semanjski and Macek, 2016). This results in increased resolution, sensitivity and fragmentation (Cain et al., 2014) and has contributed to the identification and localization of phosphosites in many species, including pathogenic bacteria. Once thought to be found only in eukaryotes, the discovery of Hanks-type family of kinases in bacteria indicates that a complex bacterial phosphorylation-mediated signaling system exists (Bakal and Davies, 2000). These serine/threonine protein kinases (STPKs) add a phosphate group to a serine/threonine, while BY-kinases add phosphate groups to Tyrosine residues. PTMs in bacteria have been linked to pathogenicity, virulence, resistance and persistence, and are vital for survival (Ge and Shan, 2011; Van Els et al., 2014). MS-based phosphoproteomic analysis has been carried out in a number of bacterial species, including *Bacillus subtilis* (Macek et al., 2007), *Escherichia coli* (Macek et al., 2008; Soares et al., 2013), *Streptococcus pneumonia* (Sun et al., 2009) *Listeria monocytogenes* (Misra et al., 2011), *Acinetobacter baumanii* (Soares et al., 2014), and *Mycobacterium tuberculosis*.

### The role of phosphorylation in *M. tuberculosis*

The bacillus *M. tuberculosis* is the causative agent of tuberculosis (TB), a leading global health crisis which has claimed millions of lives by continuing to evade clinical intervention. This is largely due to its ability to lay dormant for many years in the body, resurfacing if the hosts' immune system becomes compromised (Gengenbacher and Kaufmann, 2012). When the bacilli enter the human lung, they are ingested by alveolar macrophage cells of the human immune system. The macrophages respond by becoming acidic and exposing the pathogen to lytic enzymes, oxygenated lipids, fatty acids, and reactive oxygen and nitrogen intermediates (Schnappinger et al., 2003). In order to survive in the adverse environment of the macrophage, *M. tuberculosis* needs to react swiftly, which is possible through the regulatory mechanisms afforded by PTMs (Cain et al., 2014). Mycobacterial STPK phosphorylation has been long associated with pathogenicity (Sherman and Grundner, 2014), which has driven efforts to improve our understanding of the role of phosphorylation in *M. tuberculosis* (Prisic and Husson, 2014). Currently, there are six published manuscripts describing the phosphoproteome of a member of the Mycobacteria—*M. tuberculosis* H37Rv (Prisic et al., 2010; Kusebauch et al., 2014), *M. smegmatis*, and *M. bovis* BCG (Nakedi et al., 2015; Zheng et al., 2015), a clinical isolate of *M. tuberculosis* Beijing lineage (Fortuin et al., 2015) and a ΔpknE deletion mutant strain of *M. tuberculosis* (Parandhaman et al., 2014b). While these studies are discussed in more detail below, a summary of the methods used and relevant results for each of them is presented in Table 1. A major difficulty in comparing phosphoproteomic studies is that we cannot compare those phosphosites that were uniquely identified by each study, because it is impossible to determine whether that uniqueness is as a result of biological differences or the stochastic nature of discovery-driven MS-based proteomics. The only available study in the mycobacteria where those uniquely detected phosphosites are comparable is Nakedi et al. (2015), because the study compared two strains under the same experimental conditions. In this case, a relevant conclusion drawn by the authors is that the phosphosite patterns detected in *M. smegmatis* and *M. bovis* BCG are often species specific and these phosphorylation events are commonly occurring on entirely different peptides.

**Table 1:** **An overview of the methods used and results generated in the six currently available published studies investigating phosphorylation in the mycobacteria using mass spectrometry**.

**References**	**Strain/species**	**Fractionation method**	**Enrichment method**	**# Proteins identified**	**# Sites**	**%S**	**%T**	**%Y**	**Major conclusions**
Prisic et al., 2010	*M. tuberculosis* H37Rv	SDS PAGE	Titanium dioxide beads	301	516	40	60	-	A broad range of proteins are phosphorylated on S/T residues in *M. tuberculosis*, and may contribute to virulence.
Kusebauch et al., 2014	*M. tuberculosis* H37Rv	None	IMAC with PHOS-select iron affinity gel (Sigma) and then Titan-sphere Phos-TiO kit (GL Sciences Inc.)	232		32	64	4	Tyr phosphorylation does occur in *M. tuberculosis*, a Tyr phosphosite of PknB in is important in regulating growth and therefore may affect survival in stress conditions.
Parandhaman et al., 2014b	*M. tuberculosis* H37Rv, *M. tuberculosis*ΔPknE mutant	2D SDS PAGE	None	68	N/A	N/A	N/A	N/A	Proteins which are affected by the ΔPknE mutation under NO stress are hypothesized to contribute to pathogenicity of *M. tuberculosis*.
Nakedi et al., 2015	*M. bovis* BCG	None	TiO2 beads	203	289	35	61.6	3.1	Differences between fast and slow growing mycobacteria may be related to phosphorylation, with the ability to respond rapidly to stress associated with metabolic cost and slow growth.
	*M. smegmatis*	None	TiO2 beads	76	106	39.47	57.02	3.51	
Fortuin et al., 2015	*M. tuberculosis* clinical isolate	Strong cation exchange	TiO2 beads	214	414	38	59	3	Novel phosphorylation events were identified in this clinical strain, expanding our understanding of phosphorylation in *M. tuberculosis*.
Zheng et al., 2015	*M. bovis* BCG	SDS PAGE or offline ACN fractionation	TiO2 beads	398	659	39.5	48.7	11.8	Gel-based and gel-free phosphoproteomic analysis of *M. tuberculosis* may identify new pharmacological targets for drug development.

The identification of PTMs which contribute to the pathogenicity of *M. tuberculosis* by enhancing its ability to survive in the macrophage is of great interest to the medical community, as these represent attractive candidates for therapeutic intervention. This review will focus specifically on the use of MS-based techniques which have been used to identify phosphorylated proteins in the mycobacteria, with particular focus on the identification of phosphorylation sites which may contribute to pathogenicity and are conserved across pathogenic strains of mycobacteria.

## Comparative phosphoproteomic analysis of mycobacteria

Prisic et al. (2010) were the first to present a global view of the phosphoproteome of *M. tuberculosis* H37Rv. They used in-gel tryptic digest to proteolytically prepare samples of H37Rv lysate grown under conditions of NO stress, oxidative stress, hypoxia and using glucose, or acetate as a carbon source. A total of 152 samples were analyzed on an LTQ mass spectrometer following enrichment for phosphopeptides using titanium dioxide beads. In this manner, the authors detected a total of 506 phosphosites on 301 proteins and identified a dominant motif for *M. tuberculosis* STPKs, which was validated using synthetic peptides. This initial investigation reported 40% Ser: 60% Thr phosphorylation, and no Tyr phospho-sites—at the time of publication there was no conclusive molecular evidence for Tyr phosphorylation in *M. tuberculosis*, although there was a long-established association between Tyr phosphorylation and pathogenicity in other bacteria. (Ilan et al., 1999). Kusebauch et al. (2014) reported Tyr phosphorylation in *M. tuberculosis* for the first time, after establishing that the known *M. tuberculosis* STPKs have the capacity to phosphorylate Tyr, and then by carrying out LC MS/MS analysis on *M. tuberculosis* culture lysate enriched for phosphopeptides. In this manner, they detected 30 high-confidence Tyr phospho-sites on 17 *M. tuberculosis* proteins, contributing to a Ser: Thr: Tyr ratio of 34:62:4%. Intriguingly, these authors also found an additional 35 Tyr phosphorylation sites in publically accessible MS data from previously published proteomic studies of *M. tuberculosis*, where the assumption that there was no Tyr phosphorylation in *M. tuberculosis* had led the authors to overlook them. Subsequent MS based descriptions of the *M. tuberculosis* phosphoproteome have identified similar proportions of Tyr phosphorylation sites, some of which may be of particular importance in establishing virulence.

Recently, Nakedi et al. (2015) investigated the impact of protein phosphorylation in growth-related functions by measuring differential phosphorylation between two mycobacterial species during exponential growth phase—the fast growing, non-pathogenic, soil dwelling *Mycobacterium smegmatis* and the slow growing, attenuated strain of *Mycobacterium bovis* (BCG). BCG had consistently higher phosphorylation levels, with 289 phosphosites on 203 proteins, compared to *M. smegmatis* with 106 phosphosites found on 76 proteins. The phosphoproteins which were uniquely found in BCG were generally involved in cell growth and stress response. Even under optimal growth conditions, BCG appears to have a high level of phosphorylated stress response proteins which suggests the capability for quick on/off responses to stressors within the host, which ultimately allows the bacteria to respond rapidly and survive more effectively. The potential adaptive advantage for the pathogen may result in a fitness cost of slower growth. In a follow-up phosphoproteomic study, Zheng et al. (2015) found 659 phosphosites on 398 proteins in BCG harvested during stationary phase. The majority (40.1%) of identified phosphoproteins in this case were involved in regulation of metabolism. Again, these findings indicate that phosphorylation plays an important role in the slower metabolism of BCG, which may ultimately increase the capability for persistence within the host, and may be beneficial to the bacteria when faced with drug treatment (Evangelopoulos and McHugh, 2015).

A phosphoproteomic investigation of a hyper-virulent Beijing strain of *M. tuberculosis* by Fortuin et al. (2015) reported the identification of 414 phosphosites on 214 proteins. Of these, 252 were novel phosphosites which had not been identified in previous phosphoproteome research on the H37Rv strain. Since the capability for complex signaling is directly related to the number of phosphorylation events, it could be inferred that an increasing number of phosphorylation events may play a role in differentiating virulence in different *M. tuberculosis* strains. This is supported by findings that a highly virulent, drug resistant strain of *Acinetobacter baumannii* has almost double the number of phosphosites in comparison to the reference strain (Soares et al., 2014). It is also interesting to note that phosphorylation may be linked to drug resistance, which is of concern given the emergence of drug resistant strains of TB (Evangelopoulos and McHugh, 2015). Although the mechanism by which a bacterium might accumulate additional phosphosites is not fully understood, it is of interest that Fortuin et al. (2015) identified phosphorylated forms of 9 out of the 11 STPKs encoded by the *M. tuberculosis* genome in this hypervirulent Beijing strain (Prisic and Husson, 2014), while Prisic et al. (2010) only identified four in H37Rv.

Although our understanding of the specific activity of Mycobacterial STPKs is largely incomplete, some substrates and their downstream functions have been associated with specific kinases (Supplementary Table 1). While mass spectrometry is capable of identifying hundreds of phospho-substrates, more laborious methods are necessary to associate these substrates with their corresponding kinases. The challenges inherent in mass-spectrometry-based kinase substrate identification are discussed in more detail by Sherman and Grundner (2014). Parandhaman et al. (2014b) made use of a Δ*PknE* deletion mutant of *M. tuberculosis* to identify PknE substrates during NO stress in *M. tuberculosis*, and identified 68 phosphoproteins by combining 2D PAGE MS with phospho-serine and phospho-threonine specific antibodies. The candidate PknE substrates identified in this manner may play a role in dormancy within the macrophage, which may have implications for virulence, once again highlighting the importance of phosphorylation in allowing the bacterium to respond effectively to the host environment. Given this information, and somewhat surprisingly, there is evidence that the PknE gene is non-essential for growth of *M. tuberculosis* in culture (Sassetti et al., 2003). Indeed, in culture-based models it seems that only three of the STPKs available to *M. tuberculosis* are individually essential for growth: PknA and PknB (Wehenkel et al., 2008); and PknG, which is essential for survival of *M. tuberculosis* within the macrophage. Walburger et al. (2004) demonstrated that a strain of *M. bovis* BCG carrying an inactivated PknG gene showed no differences in growth or cell morphology in liquid medium to the wild type, whereas the mutant was unable to survive when inside a macrophage because the bacteria were no longer able to prevent lysosomal fusion. In many pathogenic bacteria, but particularly in *M. tuberculosis*, we are increasingly aware of the complexity of the host/pathogen interaction during disease progression, while being limited to culture or animal based models in our attempts to understand it^1^. The fact that *M. tuberculosis* has evolved to exist in the intracellular space is highlighted by the metabolic changes which are observed in intracellular *M. tuberculosis* (Lee et al., 2013), but we have yet to explore the consequences of phosphorylation for *M. tuberculosis in vivo*. It is conceivable that intracellular STPKs have different activity compared to culture-based systems, and high-throughput phosphoproteomic analysis of intracellular *M. tuberculosis* would allow us to better understand the role of phosphorylation in the host pathogen interaction.

### Identifying functional phosphosites for further characterization

The aim of discovery-based proteomics has been to catalog as many proteins as possible in a sample, with the intent being to better understand a biological condition or response. However, the volumes of data generated in this manner have not necessarily achieved a deeper understanding of the biological systems in question. This problem is equally as confounding, if not more so, in phosphoproteomic investigations, since it is difficult to attribute biological significance to a discreet phosphorylation event. Many of the detected phosphorylation events reported by these large-scale studies require validation before we can begin to determine their function. To this end, meta-analysis of the available data may provide some insight for future investigations. Although the above-mentioned mycobacterial phosphoproteomic studies were conducted using different MS-based methodologies, and in different strains of mycobacteria, there are discreet phosphorylation events that were commonly observed in several datasets, and are therefore unlikely to be random. A phosphopeptide which is observed in more than one study, or across several mycobacterial strains, may therefore be the best starting point for further investigation in *M. tuberculosis*. To this end, here we present a compilation of commonly detected phosphopeptides/sites which may thus be of use for future functional phosphoproteomic investigations in the mycobacteria (Supplementary Table 2).

**Figure 1:**
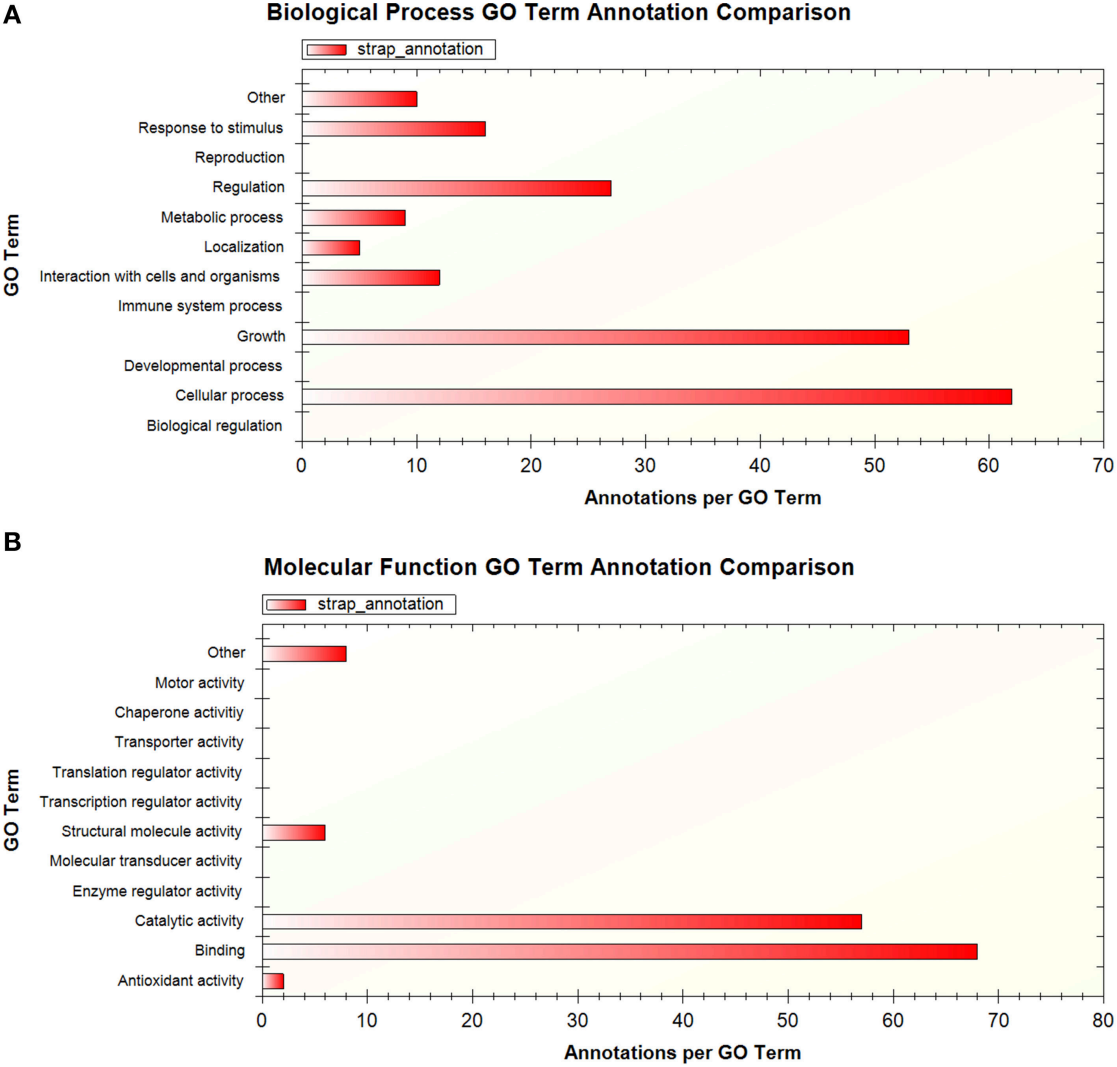
**Summary of GO terms associated with proteins that were detected in more than one phosphoproteomic dataset, shown by (A) biological process (B) molecular function**. GO analysis performed using STRAP 1.5.

A total of 194 phosphopeptides representing 148 proteins were found to be represented by two or more sets of data according to the available published supplementary information. To facilitate comparison at the protein level, the peptide sequences were matched to H37Rv protein identifiers using the Protein Information Resource (PIR) batch peptide match tool against the Uniprot H37Rv proteome (Chen et al., 2013). GO analysis of the proteins corresponding to these shared phosphopeptides using STRAP 1.5 (Bhatia et al., 2009) revealed that the associated GO terms relate to broad regulatory functions, such as the regulation of cell metabolism, cellular processes, and growth (Figures 1A,B). While this is expected given the established role of phosphorylation in metabolic regulation in bacteria (Kochanowski et al., 2015), this is also potentially noteworthy as we are only beginning to unravel the importance of metabolic regulation for intracellular *M. tuberculosis* and how this relates to virulence (Eisenreich et al., 2010). Of particular interest are incidences where the more virulent clinical strain is phosphorylated on a different residue compared to the other strains, as in the example of the AEASIETPTPVQSQR peptide of TatA, a Sec-independent protein translocase protein, which was phosphorylated on T7 and/or T9 in *M. bovis* BCG and *M. tuberculosis* H37Rv but on S4 in the clinical strain. The functional significance of this difference remains unknown and should be validated and investigated, particularly in light of the contribution of the Tat pathway to virulence in *M. tuberculosis* (Feltcher et al., 2010). It should be noted that the utility of GO analysis in mycobacteria is limited by the availability of GO annotations and other resources such as KEGG pathway representation, for which coverage is generally poor.

Differential phosphorylation of the STPKs themselves can alter their enzymatic activity, and is another possible mechanism for altered pathogenicity in *M. tuberculosis* (Chopra et al., 2003; Durán et al., 2005). Supplementary Table 2 highlights that phosphorylation is commonly detected in PknA, B, D, E, G, and H in these mycobacteria; however the differences in localized phosphosites between the virulent and less virulent strains are more pronounced in peptides corresponding to PknA, D, and G. Having already established the importance of PknA (Singh et al., 2006) and G (Walburger et al., 2004) for survival within the macrophage, the biological significance of these differences in phosphorylation and how they contribute virulence should now be ascertained. Forrellad et al. (2013) published a summary of the known virulence factors in the *M. tuberculosis* complex and their putative contribution to virulence. Cross referencing their table of results to ours identified proteins which are known virulence factors which are also commonly detected in these phosphoproteomic datasets. These proteins and their putative role in virulence in the *M. tuberculosis* complex are presented in Table 2. Included in these are some previously mentioned STPKs, as well as the proteins KatG, EspR, and IdeR.

**Table 2:** **Previously reported virulence factors in the *M. tuberculosis* complex which have been identified in more than one phosphoproteomic dataset, and their putative role in virulence**.

**Accession**	**Protein names**	**Phosphopeptides/sites**	**Putative role in virulence**	**References**
P9WI73	Serine/threonine-protein kinase PknG	**S**GPG**T**QPADAQTA**T**SA**T**VR	Unclear, possibly growth and metabolism	Nguyen et al., 2005
P9WI77	Serine/threonine-protein kinase PknE	LPVPSTHPV**S**PGTR	MAPK signaling, survival	Parandhaman et al., 2014a
P9WI79	Serine/threonine-protein kinase PknD	WSPGDSA**T**VAGPLAADSR	Unclear, plays a role in TB infection of the CNS	Be et al., 2012
P9WIE5	Catalase-peroxidase (CP) (Peroxidase/catalase) KatG	DAITSGIEVVWTNTPTK	Antioxidant	Ng et al., 2004
P9WJB7	Nucleoid-associated protein EspR	AHGLP**S**AAQQK	Transcriptional regulation	Blasco et al., 2012
P9WMH1	Iron-dependent repressor IdeR	MNELVD**T**TEMYLR	Iron dependent regulation	Banerjee et al., 2011
I6Y748	Membrane protein	DPP**T**DPNLR	Unknown	n/a

*The commonly detected phosphopeptide is indicated in bold, along with phosphosites that have been confirmed in more than one study*.

## Current state of phosphoproteomics

Currently, the mycobacterial phosphoproteome has been qualitatively described, which has identified many high confidence phosphosites. However, a complete description of the phosphoproteome should include quantitative information for the specific phosphosite in question as well as for the phosphorylated protein. In addition, most of the available phosphoproteomic data is for bacterial culture under a single condition, and yet we recognize that phosphorylation is dynamic and can change rapidly (Macek et al., 2009). In order to address this, it is important to incorporate multiple time points and experimental conditions in future phosphoproteomic investigations. These objectives are specifically challenging for mass spectrometry-based proteomics, and despite promising technological advances the mycobacterial phosphoproteome has not yet been quantitatively assessed (de la Fuente van Bentem et al., 2008). There are many available quantitative proteomic tools which are applicable in bacterial phosphoproteomics, which have been discussed in detail elsewhere (Jers et al., 2008; Macek et al., 2009), however, not all of these are suitable for the mycobacteria. SILAC, for example, has been used successfully to quantify proteins in the bacterium *Bacillus subtilis* (Ravikumar et al., 2014), but is not currently possible in *M. tuberculosis* because the available lysine deficient mutants are not viable for SILAC. Alternative quantitative methods include label-free quantitation, which has the benefit of cost effectiveness allowing for multiple time points/conditions to be explored, or dimethyl labeling, which is a promising alternative to iTRAQ and other label-based techniques (Lau et al., 2014). iTRAQ has been successfully used to quantify the phosphorylation of arginine in *B. subtilis* (Schmidt et al., 2014), and therefore is suitable for measuring S/T/Y phosphorylation in bacterial systems, but is limited by the number of samples that can be analyzed concurrently and the high cost of the iTRAQ reagents. Validation of phosphoproteomic data using targeted proteomics along with specialized analysis software such as Skyline is an exciting prospect, as these powerful tools have the capability to validate specific phosphosites as well as providing quantitative information. The absolute quantitation of modified peptides is possible through a combination of heavy-labeled AQUA peptides and selected reaction monitoring (SRM) MS, although the successful use of this strategy has not yet been reported in bacteria (Kirkpatrick et al., 2005).

## Recommendations for future research

The field of phosphoproteomics would benefit greatly from an integrated, easily accessible database containing all available information, which would facilitate meta-analysis of PTMs between multiple datasets. Furthermore, such a database for all known PTMs would allow for more in-depth analysis of the cross-talk between bacterial PTMs and how this may relate to pathogenicity, which has been discussed in a review by Soufi et al. (2012). Such a resource could provide a standardized format for reporting PTMs, as well as the opportunity to automatically update database accession numbers for modified proteins—which would in turn facilitate more meaningful comparison between different species or strains. Significant challenges exist prior to the development of such a database, including the lack of standardized reporting in currently published studies. However, the capacity to detect PTMs using MS will only increase with improving technology and the interpretation and management of the data thus generated is therefore crucial if we are to translate this into meaningful clinical applications. Through better understanding of the function of regulatory PTMs in *M. tuberculosis*, we may reveal the means to control or cure it.

## Author contributions

CA Wrote the original draft of this paper, read, and edited final draft. BC Edited draft submitted by CA, compiled data from phosphoproteomic reviews and performed meta-data analysis, put together final draft of paper. NS Supervised revisions on paper, provided editing support, and directed the topic to be covered by the review. JB Provided supervision and guidance throughout and offered editing support.

### Conflict of interest statement

The authors declare that the research was conducted in the absence of any commercial or financial relationships that could be construed as a potential conflict of interest.

## Footnotes

^1^MISSING:pmid:22320122. MISSING:pmid:22320122. (2016).
