# Supplementary material for: Mass Spectrometry Offers Insight into the Role of Ser/Thr/Tyr Phosphorylation in the Mycobacteria
Source: Front Microbiol. 2016 Feb 12;7:141. doi: 10.3389/fmicb.2016.00141 (PMC4751927; doi:10.3389/fmicb.2016.00141)
Supplement: Supplementary file 1 [file Table1.DOCX]

Supplementary Table1: Known substrates of Mycobacterial STPKs and their functions

| **Gene name** | **Rv number** | **Substrate** | **Function** | **Clade^REF^** | **Reference** |
| --- | --- | --- | --- | --- | --- |
| pknA | Rv0015c | GroEL1, KasB, Wag31 | Heat shock, Mycolic acid biosynthesis  Cell divison | I | (1) |
| pknB | Rv0014c | GarA,KasB, Rv0020c, Rv1422, Rv1747, Wag31 | Glycogen recycling, Tricarboxylic acid cycle, Mycolic biosynthesis, FHA-containing protein, Putative ABC transporter, Cell division | I | (2,3) |
| pknD | Rv0931c | GarA, GroEL1, Rv1747,  Rv0516c, Mmpl7 | Glycogen recycling, Tricarboxylic acid cycle, Heat shock protein, Putative ABC transporter | II | (4,5) |
| pknE | Rv1743 | GarA, GroEL1, KasB, Rv1747 | Glycogen recycling, Tricarboxylic acid cycle, Mycolic biosynthesis, Heat shock protein, Putative ABC transporter | II | (6) |
| pknF | Rv1746 | GarA, GroEL1, KasB, Rv0020c, Rv1747 | Glycogen recycling, Tricarboxylic acid cycle, Mycolic biosynthesis, FHA-containing protein,Putative ABC transporter | III | (7) |
| pknG | Rv0410c | GarA | Glycogen recycling, Tricarboxylic acid cycle | V | (7,8) |
| pknH | Rv1266c | embR | Tricarboxylic acid cycle | II | (9) |
| pknI | Rv2914c | EmbR/EmbR2 | Phosphotransferase, Tricarboxylic acid cycle, Glycan biosynthesis and metabolism | III | (10) |
| pknJ | Rv2088 | MyBP | Tricarboxylic acid cycle, Glycan biosynthesis and metabolism, Integral membrane protein | III | (11) |
| pknK | Rv3080c | virS | Phosphotrasferase, Tricarboxylic acid cycle  Glycan biosynthesis and metabolism | IV | (12) |
| pknL | Rv2176 | GroEL1, KasB  Rv2175 | Heat shock Protein, Mycolic acid biosynthesis | I | (13) |

**References**

1. Chaba R, Raje M, Chakraborti PK. Evidence that a eukaryotic-type serine/threonine protein kinase from Mycobacterium tuberculosis regulates morphological changes associated with cell division. *European Journal of Biochemistry* (2002) **269**:1078–1085.

2. Av-Gay Y, Jamil S, Drews SJ. Expression and characterization of the Mycobacterium tuberculosis serine/threonine protein kinase PknB. *Infection and immunity* (1999) **67**:5676–5682.

3. Young TA, Delagoutte B, Endrizzi JA, Falick AM, Alber T. Structure of Mycobacterium tuberculosis PknB supports a universal activation mechanism for Ser/Thr protein kinases. *Nature Structural \& Molecular Biology* (2003) **10**:168–174.

4. Greenstein AE, MacGurn JA, Baer CE, Falick AM, Cox JS, Alber T. M. tuberculosis Ser/Thr protein kinase D phosphorylates an anti-anti-sigma factor homolog. *PLoS Pathog* (2007) **3**:e49.

5. Peirs P, Wit L, Braibant M, Huygen K. A serine/threonine protein kinase from Mycobacterium tuberculosis. *European Journal of Biochemistry* (1997) **244**:604–612.

6. Molle V, Girard-Blanc C, Kremer L, Doublet P, Cozzone AJ, Prost J-F. Protein PknE, a novel transmembrane eukaryotic-like serine/threonine kinase from Mycobacterium tuberculosis. *Biochemical and biophysical research communications* (2003) **308**:820–825.

7. Koul A, Choidas A, Tyagi AK, Drlica K, Singh Y, Ullrich A. Serine/threonine protein kinases PknF and PknG of Mycobacterium tuberculosis: characterization and localization. *Microbiology* (2001) **147**:2307–2314.

8. Fiuza M, Canova MJ, Zanella-Cléon I, Becchi M, Cozzone AJ, Mateos LM, Kremer L, Gil JA, Molle V. From the characterization of the four serine/threonine protein kinases (PknA/B/G/L) of Corynebacterium glutamicum toward the role of PknA and PknB in cell division. *Journal of Biological Chemistry* (2008) **283**:18099–18112.

9. Sharma K, Chandra H, Gupta PK, Pathak M, Narayan A, Meena LS, D’Souza RC, Chopra P, Ramachandran S, Singh Y. PknH, a transmembrane Hank’s type serine/threonine kinase from Mycobacterium tuberculosis is differentially expressed under stress conditions. *FEMS microbiology letters* (2004) **233**:107–113.

10. Narayan A, Sachdeva P, Sharma K, Saini AK, Tyagi AK, Singh Y. Serine threonine protein kinases of mycobacterial genus: phylogeny to function. *Physiological genomics* (2007) **29**:66–75.

11. Arora G, Sajid A, Gupta M, Bhaduri A, Kumar P, Basu-Modak S, Singh Y. Understanding the role of PknJ in Mycobacterium tuberculosis: biochemical characterization and identification of novel substrate pyruvate kinase A. *PLoS One* (2010) **5**:e10772\_1–e10772\_11.

12. Kumar P, Kumar D, Parikh A, Rananaware D, Gupta M, Singh Y, Nandicoori VK. The Mycobacterium tuberculosis protein kinase K modulates activation of transcription from the promoter of mycobacterial monooxygenase operon through phosphorylation of the transcriptional regulator VirS. *Journal of Biological Chemistry* (2009) **284**:11090–11099.

13. Canova MJ, Veyron-Churlet R, Zanella-Cleon I, Cohen-Gonsaud M, Cozzone AJ, Becchi M, Kremer L, Molle V. The Mycobacterium tuberculosis serine/threonine kinase PknL phosphorylates Rv2175c: mass spectrometric profiling of the activation loop phosphorylation sites and their role in the recruitment of Rv2175c. *Proteomics* (2008) **8**:521–533.
