# Supplementary material for: Mass Spectrometry Offers Insight into the Role of Ser/Thr/Tyr Phosphorylation in the Mycobacteria
Source: Front Microbiol. 2016 Feb 12;7:141. doi: 10.3389/fmicb.2016.00141 (PMC4751927; doi:10.3389/fmicb.2016.00141)
Supplement: Supplementary file 2 [file Table2.DOCX]

Supplementary Table 2: Combined data from phosphorproteomic investigations in the Mycobacteria showing phosphosites which are shared between more than one phosphoproteomic investigation or strain/species. The strains represented here are: *M. bovis* BCG, *M. tb* H37Rv and *M. tb* SAW5527 (clinical isolate).

| **Protein accession*** | **Protein name** | **Phosphopeptide sequence** | ***M. smegmatis*** | | ***M. bovis* BCG** | ***M. tuberculosis* H37Rv** | ***M. tuberculosis* SAW5527 (Beijing)** | **Reference** |
| --- | --- | --- | --- | --- | --- | --- | --- | --- |
| I6WYU2 | Uncharacterized protein | FLL**T**G**S**AR | - | T4 | | - | S6 | (22,24) |
| I6WZG6 | 29 kDa antigen CFP29 | VVDVSDPGGPVTAAVSTGR | - | NL | | NL | - | (20,22) |
| I6WZI4 | Conserved protein | VHDDADDQQD**T**EAIAIPAHSLEFLSELPDLR | - | - | | NL | T11 | (20,24) |
| I6WZM9 | Cold-shock protein | SSALP**T**GVEALK | - | T6 | | - | T6 | (22,24) |
| I6XF31 | Conserved alanine and glycine and valine rich protein | EAAAAQAD**T**QRQAAAGVAR | - | T9 | | T9 | - | (20,22) |
| I6XF60 | Conserved alanine and arginine rich protein | SDDSSG**S**APQPAATPVPR | - | S7 | | - | S7 | (22,24) |
| I6XWB9 | Uncharacterized protein | YSSPQ**T**DFQR | - | T6 | | - | NL | (22,24) |
| I6Y231 | Polyketide synthase | ALAQYLADTLAEEQAAAPAA**S** | - | S21 | | S21 | S21 | (20,22,24) |
| I6Y2G3 | Probable phosphoglucomutase PgmA (Glucose phosphomutase) (PGM) | GRTEALADGIVVTP**S**HNPPSDGGIK | - | - | | NL | S15 | (20,22,24) |
|  |  | TEALADGIVVTP**S**HNPP**S**DGGIK | (S15) | S13, S18 | | S18 | - | (20,22,23) |
| I6Y4U9 | Conserved protein | DLLGFVDGTENP**S**GPIAIK | - | S13 | | S13 | - | (20,23) |
| I6Y748 | Membrane protein | DPP**T**DPNLR | - | T4 | | - | T4 | (22,24) |
| I6Y9Q3 | 2-methylcitrate synthase | **T**GPLAAAR | - | T1 | | - | T1 | (22,24) |
| I6YC91 | Uncharacterized protein | SDHGEIGDVEPLAD**ST**ASQAR | - | S15 | | - | S15, T16 | (23,24) |
| L0T905 | Anti-sigma-E factor RseA | AALRD**S**HPIR | - | S6 | | - | S6 | (22–24) |
| L7N653 | Probable peptidoglycan hydrolase | AALTALGMLDHQEEDL**TT**GR | - | T18 | | T18 | T17 | (20,22–24) |
| L7N686 | Uncharacterized protein | SEPRFTRDVDIVVAVANDDAAESLVR | - | NL | | NL | - | (20,22) |
| O05773 | Hydroxylase | SATQAAAATAESALGAAR | - | - | | NL | NL | (20,24) |
| O05777 | Conserved protein | **TT**PGRPLTTLDK | - | NL | | - | T1, T2 | (22,24) |
| O06194 | Conserved protein | SLPDAH**S**GS | - | S7 | | - | S7 | (22,24) |
| O06278 | Membrane protein | ALDGESPPADWVSSDRVMAVR | - | NL | | NL | - | (20,22) |
|  |  | DPL**T**GGQSVADLMAR | - | T4 | | T4 | T4 | (20,22–24) |
|  |  | LQVES**T**GGGR | - | NL | | - | T6 | (22,24) |
|  |  | TADTPPDD**S**GGLHARDPLTGGQSVADLMAR | - | S9 | | NL | - | (20,22) |
| O06396 | Conserved protein | LDPTAYGAYY**S**GPDEGPASPAERPPYR | - | S11 | | NL | - | (20,22) |
| O06404 | Possible conserved membrane protein | VQTPDDP**T**FALAR | - | T8 | | - | T8 | (22–24) |
| O07196 | Conserved protein | RIPGID**T**GR | - | T7 | | T7 | T7 | (20,22–24) |
| O07235 | Membrane protein | QPEPEVD**T**AR | - | T8 | | - | T7 | (22,24) |
| O07419 | Probable conserved Mce associated membrane protein | AAD**S**AESDAGADQ**T**GPQVK | - | S4, T14 | | NL | T14 | (20,22–24) |
| O07422 | Mammalian cell entry protein | MEDQQSA**S**GDL**T**QK | - | S8, T12 | | - | S8, T12 | (22,24) |
| O07806 | Aminoglycoside phosphotransferase | LWQAEDD**SS**R | - | - | | S9 | S8, S9 | (20,24) |
|  |  | RLWQAEDD**SS**R | - | S9, S10 | | S10 | - | (20,22) |
| O53298 | Uncharacterized protein | VTTRDIDALFSTDGPMLEAIR | - | - | | NL | NL | (20,24) |
| O53329 | Conserved protein | QFFLSG**ST**R | - | S7 | | NL | T8 | (20,22,24) |
| O53346 | Possible transmembrane cation transporter | QSGADTVVV**SS**ETAGR | - | S10, S11 | | S11 | S11 | (20,22,24) |
| O53360 | Phosphomannomutase | HTGAAAGIQITA**S**HNPATDNGYK | - | - | | S13 | NL | (20,24) |
| O53417 | Conserved protein | TEPAAATTTNASDEPA**T**GAEQAVD**T**AATPQTPEPQPIR | - | T17, T25 | | - | NL | (22,24) |
| O53521 | Long-chain-fatty-acid--CoA ligase FadD15 | NEQATTEAF**T**DGWFK | - | - | | T10 | T10 | (20,24) |
| O53578 | Methylmalonyl-CoA carboxyltransferase | TV**T**EPVLHTTAEK | - | T3 | | - | T3 | (22,24) |
| O53580 | Long-chain-fatty-acid--AMP ligase FadD32 | FDPED**T**SEQLVIVGER | - | T6 | | T6 | T6 | (20,22,24) |
| O53718 | Possible conserved secreted protein | AQPDPA**T**TVLPDPAR | - | T7 | | - | T7 | (22,24) |
| O65934 | ABC transporter ATP-binding/permease protein Rv1747 | GG**T**EAGNLA**TS**MMK | (T11) | T10 | | - | S10, T3, T10 | (22,24) |
|  |  | IPAAPP**S**GPQPR | - | S7 | | S7 | S7 | (20,22–24) |
|  |  | **T**GPTPPPPPVEQPAELGDPAHTSLFR | - | - | | NL | T1 | (20,24) |
|  |  | YPTGGQQLWPP**S**GPQR | - | S12, T3 | | S12 | S12, T3 | (20,22,24) |
| O69637 | Membrane protein | NGVPS**T**LTTIPLADPHAGPAEP**S**IGDLIK | - | S23 | | NL | T6 | (20,23,24) |
| O69654 | Uncharacterized protein | GTPAGE**T**ASFP**T**GWR | - | NL | | - | T7, T12 | (22,24) |
| O86351 | Uncharacterized protein | ARPSEP**T**NPTHHPISQVR | - | - | | NL | T7 | (20,24) |
|  |  | SYQPSEPSGPAEPVNR | - | - | | NL | NL | (20,24) |
| P71590 | FHA domain-containing protein FhaA | FEQ**S**SNLH**T**GQFR | T9 | T9 | | T9 | S4, T9 | (20,22–24) |
|  |  | GGQGQGRPDE**Y**YDDR | - | Y11 | | - | Y11 | (23,24) |
|  |  | GGYPPETGGYPPQPG**Y**PRPR | - | Y16 | | - | Y16 | (21–24) |
|  |  | HPGQGD**Y**PEQIG**Y**PDQGGYPEQR | - | Y13 | | - | Y7 | (21–24) |
| P71599 | Uncharacterized protein | GAGGSDLD**T**TR | - | NL | | - | T9 | (22,24) |
| P71658 | Putative integration host factor MihF | AQEIMTELEIAP**T**RR | - | T13 | | T13 | T13 | (20,22–24) |
| P71668 | Lipase | IAVAGD**S**AGGTIAAVIAQR | - | S7 | | S7 | - | (20,22,23) |
| P71977 | Probable transcriptional regulatory protein | **S**GGIQVIAR | - | S1 | | S1 | S1 | (20,22–24) |
| P95017 | Membrane protein | AAD**T**DVF**S**AVR | - | T4 | | T4 | S8, T4 | (20,22–24) |
|  |  | AADTDVFSAVRADDSPTGEMQVAQPEAQTAAVATVER | - | NL | | NL | - | (20,22) |
|  |  | ADDSP**T**GEMQVAQPEAQ**T**AAVATVER | - | T6, T18 | | T18 | T6, T18 | (20,22–24) |
|  |  | EAP**T**EVIR | - | T4 | | - | T4 | (22,24) |
|  |  | TTESDTP**T**EVIR | - | T8 | | T8 | - | (20,23) |
| P95034 | Putative ferredoxin reductase | IAAGAPIAEVLDQ**T**QA | - | T14 | | - | T14 | (22,24) |
| P95172 | NADH:ubiquinone oxidoreductase subunit J | GADGLQTPSPGAVSGSLEGGA**S** | - | S22 | | - | S22 | (22,24) |
| P95197 | Phosphoribosylglycinamide formyltransferase 2 | AAGHQVQPQ**T**GGVSPR | - | T10 | | T10 | T10 | (20,22,24) |
| P96267 | Alpha/beta hydrolase | GLAEGPLIAGGH**SY**GGR | - | S13, Y14 | | S13 | S13, Y14 | (20,22–24) |
| P96356 | Uncharacterized protein | GIPDSRTSKDFDTVAR | - | NL | | NL | - | (20,22) |
| P96409 | Probable conserved membrane protein | GGFEEPVPGAEAE**T**EK | - | - | | T14 | T14 | (20,24) |
|  |  | GGFEEPVPGAEAE**T**EKLPTQRPDFPR | - | T14 | | T14 | - | (20,23) |
| P96805 | Membrane protein | RGELDSGTSTPA**T**NYGS**S** | - | T13 | | - | S18 | (22,24) |
| P9WF15 | Putative antitoxin VapB49 | LAALDS**T**D**T**LER | - | - | | T7 | T7, T9 | (20,24) |
| P9WFK9 | UPF0234 protein Rv0566c | AFEAGEPQA**S**GK | - | S10 | | - | S10 | (22–24) |
| P9WFZ1 | tRNA (adenine(58)-N(1))-methyltransferase TrmI | SA**T**GPFSIGER | - | - | | NL | T3 | (20,24) |
| P9WG39 | Acetolactate synthase large subunit IlvG | STD**T**APAQTMHAGR | - | T4 | | T4 | T4 | (20,22–24) |
| P9WG47 | DNA gyrase subunit A | FPNLLANG**S**GGIAVGMATNIPPHNLR | - | S9 | | NL | - | (20,23) |
| P9WGA1 | Sec-independent protein translocase protein TatA | AEA**S**IE**T**P**T**PVQSQR | - | T7, T9 | | T7, T9 | S4 | (20,22–24) |
|  |  | VDPSAASGQDS**T**EARPA | - | T12 | | T12 | T12 | (20,22,24) |
| P9WGC3 | Uncharacterized SufE-like protein Rv3284 | LHFSAPAEAP**TT**R | - | - | | NL | T11, T12 | (20,24) |
| P9WGD5 | Single-stranded DNA-binding protein | SGGFGSGSRPAPAQTSSASGDDPWGSAPA**S**GSFGGGDDEPPF | - | S30 | | NL | - | (20,22) |
| P9WGD9 | Signal recognition particle receptor FtsY | ID**TS**GLPAVGDDA**T**VPR | NL | S4, T3 | | T3 | S4, T3, T14 | (20,22–24) |
| P9WGM1 | Transcriptional regulatory protein PrrA | VLVVDDD**S**DVLASLER | - | S8 | | S8 | - | (20,22) |
| P9WGM3 | Probable transcriptional regulatory protein pdtaR | **T**GPTTDADAAVPR | - | NL | | - | T1 | (22,24) |
| P9WGN7 | Protein translocase subunit SecE | AAGADADVDVEEPSTAASEATGVAKDDSTTK | - | NL | | NL | - | (20,22) |
| P9WGN9 | Protein translocase subunit SecF | NSGSPAGSEDASTDGGEQPAAADEQ**S**LVGITQASSQSAPR | - | S26 | | NL | - | (20,22) |
|  |  | TGRDDEATSAVEL**T**EATESAVAR | - | NL | | T14 | - | (20,22) |
| P9WGS1 | Uncharacterized oxidoreductase Rv1543 | GSESQTD**T**SELDKR | - | T8 | | - | NL | (22,24) |
| P9WGV3 | Adenosylhomocysteinase | GVTEET**TT**GVLR | - | T8 | | T8 | T7, T8 | (20,22–24) |
|  |  | **T**GNLVTK | - | NL | | - | T1 | (22,24) |
| P9WH07 | Ribosomal RNA small subunit methyltransferase A | APDISGHASA**S** | - | S11 | | - | S11 | (22,24) |
| P9WH35 | 30S ribosomal protein S4 | SLN**T**VPFQIAR | - | T4 | | T4 | - | (20,22) |
| P9WH37 | 30S ribosomal protein S3 | AAGGEEAAPDAAAPVEAQSTE**S** | - | NL | | - | S22 | (22,24) |
| P9WH43 | 30S ribosomal protein S1 | GADDQSSASSAPSEK | - | NL | | - | NL | (22,24) |
| P9WH45 | 30S ribosomal protein S19 | HVPVFVTE**S**MVGHK | - | NL | | - | S9 | (22,24) |
| P9WHB7 | 50S ribosomal protein L24 | **S**GGIVTQEAPIHVSNVMVVDSDGKPTR | - | S1 | | - | S1 | (23,24) |
| P9WHP5 | 35 kDa protein | YANAIGSAELAE**S**SVQGR | - | S13 | | - | S13 | (22,24) |
| P9WHQ1 | Uncharacterized RNA pseudouridine synthase Rv1711 | LDAD**T**EGLMLLTNDGELAHR | - | T5 | | NL | - | (20,22) |
| P9WHT3 | Probable cytosol aminopeptidase | T**T**EPG**Y**LSPSVAVATSMPK | - | T2 | | T2, Y6 | NL | (20–22,24) |
| P9WHW5 | PP2C-family Ser/Thr phosphatase | ITPEEAH**S**HPQR | - | S8 | | - | S8 | (22–24) |
| P9WI55 | Inorganic pyrophosphatase | HFFVH**Y**K | - | NL | | - | Y6 | (22,24) |
| P9WI71 | Serine/threonine-protein kinase PknH | LTQLG**T**AVGTWK | - | T6 | | - | T6 | (22,24) |
| P9WI73 | Serine/threonine-protein kinase PknG | **S**GPG**T**QPADAQTA**T**SA**T**VR | - | - | | NL | S1, T5, T14, T17 | (20,24) |
| P9WI77 | Serine/threonine-protein kinase PknE | LPVPSTHPV**S**PGTR | - | NL | | - | S10 | (22,24) |
| P9WI79 | Serine/threonine-protein kinase PknD | WSPGDSA**T**VAGPLAADSR | - | - | | T8 | S6, S17, T8 | (20,24) |
| P9WI81 | Serine/threonine-protein kinase PknB | AIADSGNSV**T**Q**T**AAVIGTAQYLSPEQAR | T10, T12 | T10, T12 | | T12 | T10, T12 | (20,22,24) |
|  |  | TSLLSSAAGNL**S**GPR**T**DPLPR | - | T16 | | T16 | S12, T16 | (20,22–24) |
| P9WI83 | Serine/threonine-protein kinase PknA | AAPAAIP**S**G**TT**AR | (T6, T12) | - | | NL | S8, T10, T11 | (20,24) |
|  |  | RPFAGDGAL**T**VAMK | - | T10 | | T10 | T10 | (20,22,24) |
| P9WIE5 | Catalase-peroxidase | DAITSGIEVVWTNTPTK | - | S5 | | - | T4 | (23,24) |
| P9WII9 | Pyridoxal 5'-phosphate synthase subunit PdxS | MDPAGNPA**T**G**T**AR | - | NL | | T9 | T11 | (20,22,24) |
| P9WIU9 | Transcription termination/antitermination protein NusG | TTFDGD**T**SAGEAVDLTEANAFQDAAAPAEEVDPAAALK | - | T7 | | NL | - | (20,22) |
| P9WJ07 | Antitoxin Rv0909 | AGEFVDEQ**T**QGNY**S**DAIHK | - | T9 | | - | S14, T9 | (22,24) |
| P9WJ95 | Isoniazid-induced protein IniC | STLVNALVGDDIAPTDA**T**EATR | - | NL | | T18 | - | (20,22) |
| P9WJ97 | Isoniazid-induced protein IniB | AM**T**SAGLIDIAPHQISSVAANVVPGLNLGAGDPM**S**GLR | - | S35 | | T3, S35 | - | (20,22) |
| P9WJA9 | Glycogen accumulation regulator GarA | DQ**T**SDEV**T**VE**TT**SVFR | - | - | | T11 | T3, T8, T11, T12 | (20,23,24) |
|  |  | TDMNPDIEKDQTSDEVTVE**T**TSVFR | - | T20 | | T20 | - | (20,22–24) |
| P9WJB7 | Nucleoid-associated protein EspR | AHGLP**S**AAQQK | - | S6 | | - | S6 | (22,24) |
| P9WJB9 | ESX-1 secretion-associated protein EspL | **S**MDELDPHVAR | - | S1 | | - | S1 | (22–24) |
| P9WJC5 | ESX-1 secretion-associated protein EspI | LRAEEA**S**GAQLAPG**T**EPSPAPLGQPR | - | - | | S7, T15 | S7, T15 | (20,24) |
|  |  | VA**T**GP**S**IQAR | - | - | | T3 | S6, T3 | (20,24) |
|  |  | VHPDLAAQHAAAQPDSITAATTGGR | - | - | | NL | T18 | (20,24) |
| P9WJG3 | RNA polymerase-binding transcription factor CarD **/†† | AETILDEVLAAA**S** | - | S13 | | - | S13 | (22,24,25) |
| P9WJK3 | Probable peptidoglycan biosynthesis protein MviN | GGSLQEVAD**T**SPSPVGAIR | - | T10 | | T10 | - | (20,22) |
|  |  | SASTLLNLMQQATAVADR**T**EVLGPIDEAPVSAAPR | - | T19 | | T19 | - | (20,22) |
|  |  | SGLAPAERDTAGQPIEPADIDRDIPFQISAVAAR | - | T10 | | NL | - | (20,22) |
| P9WJT1 | Probable transport accessory protein MmpS3 | ASGNHLPPVAGGGDKLPSDQ**T**GE**T**DAYSR | - | T21, T24 | | T21, T24 | NL | (20,22,24) |
|  |  | A**YS**APE**S**EHV**T**GGP**Y**VPADLR | - | S3, T11, Y15 | | NL, Y2, Y15 | S3, S7, T11, Y15 | (20–22,24) |
|  |  | **S**GPNPPGREPDEPE**S**EPVSDTGDER | - | S15 | | - | S1 | (22,24) |
| P9WJV5 | Trehalose monomycolate exporter MmpL3 | MQIGSSTEPP**T**TR | - | T11 | | T11 | - | (20,22) |
|  |  | SDGDDSEAA**T**EQLNAR | - | T10 | | T10 | T10 | (20,22–24) |
|  |  | SSPASSPELTPALEATAAPAAPSGASTTR | - | NL | | NL | NL | (20,22,24) |
|  |  | STDAAGDPAEP**T**AALPIIR | - | T12 | | T12 | T12 | (20,23,24) |
|  |  | SVQSPASTPPP**T**PTPPSAPSAGQ**T**R | - | NL, T12 | | T12, T24 | - | (20,22,23) |
| P9WKE9 | Guanylate kinase | SVGEGPD**T**KPTAR | - | T8 | | T8 | T8 | (20,22–24) |
| P9WKF5 | Adenylate kinase | ADD**T**DDVILNR | - | T4 | | - | T4 | (22,24) |
|  |  | LGIPQI**S**TGELFR | - | - | | S7 | S7 | (20,24) |
| P9WKK3 | Translation initiation factor IF-1 | VLAHI**S**GK | - | S6 | | - | S6 | (22–24) |
| P9WKM1 | Uncharacterized protein Rv0966c | ASD**T**DRIQIAQLLAYAAEQGR | T4 | T4 | | NL | NL | (20,22,24) |
| P9WKP5 | Uncharacterized protein Rv0898c | KPTDSE**T**LAHIR | - | T7 | | - | NL | (23,24) |
| P9WKT7 | Putative DNA-binding protein Rv0500A | D**T**GFVEGQQAK | - | T2 | | - | T2 | (22,24) |
| P9WKU3 | Uncharacterized protein Rv0497 | RGDSDAITVAEL**T**GEIPIIR | - | - | | T13 | T13 | (20,24) |
|  |  | **T**GPHPETESSGNR | - | T1 | | - | T1 | (22,24) |
| P9WKV7 | Uncharacterized protein Rv0479c | PGDQGPLARPPASSEA**S**TGR | - | NL | | NL | S17 | (20,22,24) |
| P9WLI9 | Uncharacterized protein Rv2197c | MAEAEPA**T**RP**T**GASVR | - | T8, T11 | | T8, T11 | T8 | (20,22,24) |
| P9WLZ3 | Uncharacterized protein Rv1367c | IA**S**M**T**KPVTVAAAMSLVDEGK | - | S3, T5 | | - | - | (22,23) |
| P9WLZ7 | Uncharacterized protein Rv1364c | SGIAPVTIDLSAVTHLG**S**AGVGALAAACDR | - | S18 | | S18 | - | (20,22) |
| P9WMA7 | Uncharacterized protein Rv0007 | AA**T**GPGRIPDAGDPPPWQR | - | T3 | | T3 | - | (20,22) |
|  |  | FISGASAPV**T**GPAAAVR | - | T10 | | T10 | T6, T10 | (20,22,24) |
|  |  | TPQPDPDASLGCGDGSPAEAYASELPDL**S**GPTPR | - | S29 | | S29 | - | (20,22) |
| P9WMB5 | Phosphatidylinositol mannoside acyltransferase | NAFD**T**GAR | - | T5 | | - | T5 | (22,24) |
| P9WMH1 | Iron-dependent repressor IdeR **/††† | MNELVD**T**TEMYLR | T7 | T7 | | - | - | (22,23,25) |
| P9WMJ9 | Chaperone protein DnaK | AALGG**S**DI**S**AIK | - | S6 | | - | S9 | (22–24) |
| P9WMN5 | Protein Rv2204c | TLDGDQ**T**AEFGGVR | - | T7 | | T7 | T7 | (20,23,24) |
| P9WMR1 | Probable helicase HelY | AVFATETLALGINMPAR | - | NL | | NL | - | (20,23) |
| P9WMT5 | Protein GrpE | **T**DGNQKPDGN**S**GEQVTVTDK | - | S11, T1 | | - | S11 | (22–24) |
|  |  | TDGNQKPDGN**S**GEQVTV**T**DKR | - | S11 | | NL | T18 | (20,23,24) |
| P9WMT9 | Transcription elongation factor GreA *** | **T**DTQVTWLTQESHDR | - | T1 | | - | NL | (23,24) |
| P9WMU1 | Cell wall synthesis protein Wag31 | G**S**AAPVD**S**NADAGGFDQFNR | - | S8 | | - | S2 | (22,24) |
| P9WMU5 | Putative gluconeogenesis factor | DVGASEPPVAA**T**QEIPIDGGRPR | - | T12 | | T12 | T12 | (20,23,24) |
| P9WN27 | Glutamate-ammonia-ligase adenylyltransferase | ELNYV**S**DVDVIFVAER | S6 | S6 | | S6 | - | (20,22) |
| P9WN41 | Phosphoglucosamine mutase | TAVEQAAAELGD**T**GR | NL | T13 | | T13 | T13 | (20,22,24) |
| P9WNA1 | Cell division protein FtsQ | VADDAADEEAV**T**EPLATESK | - | T12 | | T12 | T12 | (20,23,24) |
|  |  | VADDAADEEAV**T**EPLATESKDEPAEHPEFEGPR | - | T12 | | T12 | - | (20,22,23) |
| P9WNA3 | DNA translocase FtsK | EYADDFEDFADFDGDDAD**T**VEVAR | - | T19 | | - | T19 | (22,24) |
|  |  | SGAI**T**APLGSQR | - | T5 | | T5 | T5 | (20,22,24) |
| P9WNN1 | Elongation factor Tu | NMI**T**GAAQMDGAILVVAATDGPMPQ**T**REHVLLAR | - | T4 | | T26 | - | (20,23) |
|  |  | VLHDKFPDLNE**T**K | - | T12 | | - | T12 | (22,24) |
| P9WNQ3 | ESX-3 secretion system protein eccD3 | **S**GTVMQIVR | - | NL | | - | S1 | (22,24) |
| P9WNV9 | Chaperone protein DnaJ 1 | **T**GGTTIGDLFGGLFGR | - | T1 | | T1 | - | (20,22) |
| P9WNX3 | D-3-phosphoglycerate dehydrogenase | **S**ATTVDAEVLAAAPK | - | S1 | | S1 | S1 | (20,23,24) |
| P9WNY1 | Probable aldehyde dehydrogenase | VAFTGETT**T**GR | - | - | | T9 | NL | (20,24) |
| P9WP67 | Probable cytochrome c oxidase subunit 3 | TSAVG**TS**G**T**AI**T**SR | T12 | T6, T12 | | NL | S7, T9 | (20,22,24) |
| P9WP71 | Probable cytochrome c oxidase subunit 1 | HHDEPAMVT**SS** | - | S10 | | - | S11 | (22,24) |
| P9WPB5 | Cyclopropane mycolic acid synthase 2 | TSQGDTTSGTQLKPPVEAVR | - | - | | NL | NL | (20,24) |
| P9WPC5 | ATP-dependent Clp protease proteolytic subunit 1 | SN**S**QGLSL**T**DSVYER | - | - | | T9 | S3, T9 | (20,24) |
| P9WPD1 | Chaperone protein ClpB | LLDRLPQATGAS**T**QPQLSR | - | T13 | | T13 | - | (20,22) |
|  |  | LPQATGAS**T**QPQLSR | - | - | | NL | T9 | (20,24) |
| P9WPD5 | Citrate synthase 1 | ADTDD**T**ATLR | - | - | | T6 | T6 | (20,24) |
| P9WPE5 | 10 kDa chaperonin *** | DVLAVV**S**K | - | S7 | | - | S7 | (22–25) |
|  |  | ILVQANEAET**TT**A**S**GLVIPDTAK | NL | S14, T11, T12 | | S14, T12 | - | (20,22,23,25) |
| P9WPE7 | 60 kDa chaperonin 2 | AMLQDMAIL**T**GGQVI**S**EEVGLTLENADLSLLGK | - | S16, T10 | | S16, T10 | - | (20,23) |
|  |  | AVEKV**T**ETLLK | - | NL, T6 | | - | - | (22,23) |
|  |  | EQIAA**T**AAI**S**AGDQSIGDLIAEAMDK | - | T6 | | S10, T6 | - | (20,22) |
|  |  | KWGAP**T**I**T**NDGV**S**IAK | - | S13, T6, T8 | | - | - | (22,23) |
|  |  | WGAPTI**T**NDGV**S**IAK | - | S12 | | - | T7 | (23,24) |
| P9WPR9 | Uncharacterized protein Rv1488 | HAADGDDAEVAGWFSTD**T**DPSIAR | - | T18 | | T18 | - | (20,22) |
| P9WPZ3 | Arginine biosynthesis bifunctional protein ArgJ | TDLAG**T**TR | - | T6 | | - | T6 | (22,24) |
| P9WQ13 | Probable endonuclease 4 | AA**T**LPIYVHAPYLINLA**S**ANNR | - | S18, T3 | | - | - | (22,23) |
| P9WQA5 | Uncharacterized oxidoreductase Rv2971 | LATPDQGF**T**R | - | T9 | | - | T9 | (22,24) |
| P9WQB3 | 2-isopropylmalate synthase | HA**S**DPV**T**IASPAQPGEAGR | - | S3, T7 | | - | - | (22,23) |
| P9WQF3 | Meromycolate extension acyl carrier protein | AKIE**S**ENPDAVANVQAR | - | S5 | | S5 | - | (20,23) |
| P9WQF7 | Probable acyl-CoA dehydrogenase FadE10 | AQQTQV**T**EEQAR | - | T7 | | - | T7 | (22,24) |
| P9WQM9 | L-asparagine permease 1 | LGH**T**GPFPAVANPPVR | - | T4 | | T4 | T4 | (20,22,24) |

*** Uniprot accession for *M. tb* H37Rv**

**NL: not localised/ambiguous residues**

**- not found**

**() localisation in brackets is correct for *M. smegmatis*, which has slightly different peptide sequence to *M. tb***

**** higher in ΔpknE mutant in Middlebrook medium (Parandhaman *et. al.* 2014)**

***** absent in ΔpknE mutant in Middlebrook medium (Parandhaman *et. al.* 2014)**

**†† higher in ΔpknE mutant under NO stress (Parandhaman *et. al.* 2014)**

**††† absent in Rv under NO stress (Parandhaman *et. al.* 2014)**
